# The Role of Immune Modulation in Pathogenesis of IgA Nephropathy

**DOI:** 10.3389/fmed.2020.00092

**Published:** 2020-03-24

**Authors:** Sheng Chang, Xiao-Kang Li

**Affiliations:** ^1^Institute of Organ Transplantation, Tongji Hospital, Tongji Medical College, Huazhong University of Science and Technology, Key Laboratory of Organ Transplantation, Ministry of Education NHC Key Laboratory of Organ Transplantation Key Laboratory of Organ Transplantation, Chinese Academy of Medical Sciences, Wuhan, China; ^2^Division of Transplantation Immunology, National Research Institute for Child Health and Development, Tokyo, Japan; ^3^Department of Hepatobiliary and Pancreatic Surgery, The First Affiliated Hospital of Zhengzhou University, Zhengzhou, China

**Keywords:** glomerular mesangium, IgA nephropathy, immunopathogenesis, mucosal immune, innate immunity, adaptive immunity

## Abstract

IgA nephropathy (IgAN) is the most prevalent primary glomerulonephritis worldwide, with diverse clinical manifestations characterized by recurrent gross hematuria or microscopic hematuria, and pathological changes featuring poorly O-galactosylated IgA1 deposition in the glomerular mesangium. Pathogenesis has always been the focus of IgAN studies. After 50 years of research, most scholars agree that IgAN is a group of clinicopathological syndromes with certain common immunopathological characteristics, and multiple mechanisms are involved in its pathogenesis, including immunology, genetics, and environmental or nutritional factors. However, the precise pathogenetic mechanisms have not been fully determined. One hypothesis about the pathogenesis of IgAN suggests that immunological factors are engaged in all aspects of IgAN development and play a critical role. A variety of immune cells (e.g., dendritic cells, NK cells, macrophages, T-lymphocyte subsets, and B-lymphocytes, etc.) and molecules (e.g., IgA receptors, Toll-like receptors, complements, etc.) in innate and adaptive immunity are involved in the pathogenesis of IgAN. Moreover, the abnormality of mucosal immune regulation is the core of IgAN immunopathogenesis. The roles of tonsil immunity or intestinal mucosal immunity, which have received more attention in recent years, are supported by mounting evidence. In this review, we will explore the latest research insights on the role of immune modulation in the pathogenesis of IgAN. With a better understanding of immunopathogenesis of IgAN, emerging therapies will soon become realized.

## Introduction

IgA nephropathy (IgAN) is a clinicopathological syndrome, with diverse clinical manifestations characterized by repeated episodes of gross or microscopic hematuria, and pathological changes featuring IgA1 deposition in the glomerular mesangium, mesangial cell proliferation, and matrix expansion (1). IgAN is the most common primary glomerulonephritis worldwide, and it causes 25–50% of patients to develop end-stage renal disease (ESRD) within 20 years of diagnosis and shortens life expectancy by 10 years, although the course usually evolves gradually (2). The incidence of IgAN is influenced by region, ethnicity, and race; the highest incidence is in Asia, where it accounts for up to 60% of glomerular disease, which is significantly more than the incidence in Europe (30%) and America (10%) (3). Available data confirm that the prevalence of IgAN varies among different races and that IgAN has a high tendency for family aggregation. Genetic factors are thought to be related to the occurrence and development of IgAN, although differences in the strategies and techniques for renal biopsy in different countries or regions should be considered. More specifically, the histopathological manifestations of IgAN based on characteristic IgA1, which is immunoprecipitated in the glomerulus alone or concomitant with immunoglobulin, may help to elucidate the autoimmune disease properties of IgAN. Immunological events play a decisive role in the pathogenesis of IgAN.

**Figure 1:**
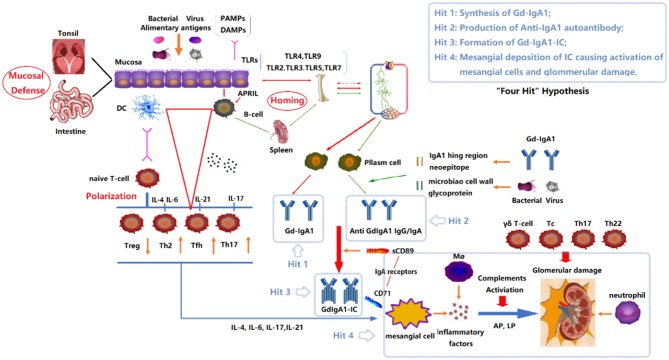
“Four Hits” Hypothesis of IgA Nephropathy. In individuals with a genetic predisposition to IgA nephropathy, infection, or other events destroy the mucosal barrier defense function. Chronic stimulation such as pathogenic microbial or alimentary antigens are taken up by antigen-presenting cells, thereby activating B cells, and differentiating into plasma cell secreted IgA in T-cell-dependent or non-dependent manners. Due to the abnormal regulation of mucosal-bone marrow axis, the mis-expression of homing receptors on the surface of B/plasm cells leads to increased synthesis of poorly glycosylated IgA1 (Gd-IgA1) and self-aggregation to form aggregated Gd-IgA1 (Hit 1). The aberrant exposure of GalNAc of Gd-IgA1 as antigen stimulates B cells to differentiate into plasma cells and synthesizes anti-Gd-IgA1 autoantibodies (Hit 2). The Gd-IgA1 immune complex (Gd-IgA1-IC) is formed by anti-Gd-IgA1 autoantibodies binding to Gd-IgA1 along with soluble sCD89 (Hit 3). Macromolecular Gd-IgA1-IC binds to the IgA receptor (CD71) expressed on mesangial cells and deposits on the glomerular mesangium. Subsequently, the mesangial cells release various inflammatory factors (cytokines, chemokines, growth factors, etc.) under the stimulation of ICs, attracting and recruiting multiple subsets of T cells, macrophages, neutrophils infiltration, and activating the lectin pathway and alternative pathway of the complement system. Synergistic effects lead to glomerular injuries such as mesangial hyperplasia, matrix expansion, and interstitial fibrosis (Hit 4). DC, Dendritic cell; TLRs, Toll like receptors; APRIL, a proliferation-inducing ligand; DAMPs, damage-associated molecular patterns; PAMPs, pathogen-associated molecular patterns; Gd-IgA1, poorly glycosylated IgA1/Galactose-deficient IgA1; AP, The alternative pathway; LP, The lectin pathway.

The most widely accepted hypothesis about the pathogenesis of IgAN has been known as a “four-hit” hypothesis (Figure 1) (4). That is, the development of IgAN requires at least four processes (called “hits”): (1) increased synthesis of poorly O-galactosylated IgA1 (also called Galactose-deficient IgA1, Gd-IgA1) in circulation; (2) production of autoantibodies against Gd-IgA1; (3) formation of immune complexes containing pathogenic O-galactosylated IgA1; and (4) mesangial deposition of these immune complexes activating mesangial cells and subsequently impairing glomeruli. In fact, multiple immune cells and many immune molecules in the immune system participate in the pathogenesis of IgAN through diverse mechanisms. This review focuses on the immune modulation of IgAN and summarizes the mechanisms associated with its pathogenesis.

## Role of Poorly O-Galactosylated IgA1 and IgA Receptors in IgAN

Abundant clinical and laboratory evidence show that both innate and adaptive immunity play important roles in various aspects of the immunological pathogenesis of IgAN. It is generally agreed that IgAN does not originate from a single “hit” of pathogenic disease, but from the result of multiple consecutive but distinct “hits” of different pathogenic diseases. As mentioned in the Introduction, the first hit is that individuals with a genetic predisposition develop aberrant immune responses to common and environmental causes, which leads to an increase of poorly O-galactosylated IgA1 in the serum. As previously reported (5), IgA accounts for about 15% of total serum immunoglobulins in humans and it is mainly present in the mucosal area (i.e., alimentary, respiratory, and urogenital tracts), but is also found in the blood. Structurally, IgA exists in two isotypes, IgA1 and IgA2, each of which can be divided into monomeric (mIgA) and polymeric (pIgA, mainly dimeric) forms. Based on location, it can also be categorized as serum IgA or secretory IgA (SIgA). Monomeric IgA is found predominantly in the serum, most of which is produced in the bone marrow. SIgA is mainly present in the mucosal areas and secretions, whereas PIgA is present at low levels in serum. The pIgA1 components are diverse, including dimeric IgA, SIgA, and IgA immune complexes (6). The primary function of SIgA on the mucosa and secretions is to neutralize toxins and prevent the infiltration and invasion of microbes (commensals and pathogens) through the mucosal epithelial barrier, thereby preventing systemic infection and maintaining a physiologically essential symbiotic relationship with commensals (7). The difference between human IgA1 and IgA2 lies only in the absence of 13 amino acids in the hinge region of IgA2. The hinge region of IgA1, however, consists of unusual repeated sequences rich in proline, serine, and threonine, with serine-containing O-linked oligosaccharide chains (8). The O-glycosylation consists of a core N-acetylgalactosamine (GalNAc), which is extended with β-1,3-linked Gal or further with sialic acid in α-2,3 and/or α-2,6 linkage. However, in the peripheral blood and renal tissues of patients with IgAN, the glycosylation of IgA1 molecules often lacks galactose molecules (Gal), resulting in the formation of monosaccharide side chains (i.e., aberrant glycosylation) containing only a single GalNAc, which may be related to the impaired function of β-1,3-galactosyltransferase. Altered glycosylation favors self-aggregation of IgA1 (9); in addition, aberrant glycosylation results in GalNAc exposure and is recognized by normal IgG and IgA1 antibodies in the body to form immune complexes (10). IgA immune complexes, which are composed of aberrantly glycosylated IgA1, may escape clearance from the liver and preferentially deposit in the kidney due to enhanced lectin reactivity with fibronectin, laminin, and collagen in the glomerular mesangial matrix.

Although aberrant IgA1 levels have been observed in peripheral blood and kidney biopsies from patients with IgAN, the source of this poorly O-galactosylated IgA1 has been of concern and several clinical observations have generated interest. Time-zero renal transplant biopsies were performed in 510 renal transplant donors in a controlled study from Japan (11). They found that mesangial IgA deposition was present in 16.1% of donor kidneys with no statistically significant difference between living donors and cadaveric donors or between related donors and non-related donors. IgA mesangial deposition often occurs in untreated celiac disease, and although IgA appears to be deposited, it rarely induces clinically significant glomerulonephritis. Another considerable phenomenon associated with kidney transplantation is IgA deposition. This frequently recurs in the allograft, with published recurrence rates of 10–53%, and recurrence tends to occur in the late phase of the post-transplant period. Additionally, after the kidney of a subclinical IgAN donor is implanted into a non-IgAN recipient, IgA deposits were removed from the kidney within a few weeks. The above data suggest that IgA1 immune complexes in glomerular mesangium of patients with IgAN are a result of deposition as opposed to *in situ* synthesis (12). Moreover, IgA deposition alone is not necessarily the main cause of kidney damage, and sometimes these subclinical IgA deposits are cleared and alleviated spontaneously (13).

To identify the molecular basis for aberrant IgA1 levels, an *in vitro* model analyzed the pathway of IgA1-producing cells from peripheral blood cells of patients with IgAN. The results showed that β-1,3-galactosyltransferase activity decreased, and N-acetylgalactosamine-specific α-2,6-sialyltransferase activity increased in IgA1-producing cells of patients with IgAN. Consequently, the data suggest that premature sialylation may contribute to abnormal IgA1 glycosylation in IgAN (14).

It has been demonstrated that aberrant glycosylation of IgA1, irrespective of O-glycans or N-glycans (15), significantly contributes to the pathogenesis of IgAN. An *in vitro* study using a model of human mesangial cells found that the aggregated IgA1 with underglycosylation from patients with IgAN bound glomerular mesangial cells with more high affinity and could induce profibrotic cytokine production and proliferation in mesangial cells, and the effects were more significant compared to aggregated IgA1 from a healthy control group (16). In addition, aberrantly glycosylated IgA1 acts as an autoantigen and forms IgG autoantibodies against Gal-deficient IgA1 molecules; these bind to the aberrantly glycosylated IgA1 molecules to form circulating immune complexes that are deposited in the glomerular mesangium, thereby causing kidney damage (17).

In recent years, the role of receptor-ligand binding between IgA1 circulating immune complexes and IgA receptors of mesangial cells in the pathogenesis of IgAN has attracted much attention. The deposition of circulating immune complexes in the renal mesangium mainly depends on its high affinity to IgAl receptors on mesangial cells. After binding, mesangial cells are induced to secrete inflammatory factors and activate complements, leading to pathological changes and clinical symptoms of IgAN. It is generally believed that the IgA receptor family comprises polymeric Ig receptors, involved in epithelial transport of IgA/IgM, myeloid-specific IgA Fc receptors (FcαRI or CD89), Fcα/μR, and some other IgA receptors. These are asialoglycoprotein receptors, transferrin receptors (TfR1 or CD71), FCRL4, and DC-SIGN/SIGNR1, which are involved in IgA catabolism and tissue IgA deposition (18). Recently, another new IgA receptor has been identified, namely β-1,4-galactosyltransferase 1 (β-1,4-GalT1). It is expressed constitutively by the human mesangial cells and its levels are increased in patients with IgAN. This receptor plays an important role in the deposition and clearance of mesangial IgA (19).

However, CD89 and CD71 are currently considered more important than other receptors and have accordingly attracted more attention. In a transgenic murine model co-expressing human IgA1 and CD89, the mice exhibited IgA1-sCD89 complexes in circulation, mesangial deposits, and subsequent glomerular inflammation in a similar manner as IgAN patients, whereas mice expressing only IgA1 did not experience mesangial impairment or renal dysfunction. However, following sCD89 injection, sCD89 and IgA1 deposits were detected in the mesangium of IgA1-expressing mice (20). It is proposed that the pathogenic complexes containing polymeric Gd-IgA1 could facilitate IgA binding to CD89 on blood monocytes (21). The cleavage of FcRc-less CD89 would produce soluble CD89/Gd-IgA1 complexes, that would be deposited in the mesangium via IgA1-sCD89 complexes binding to CD71. The interaction of sCD89 with CD71 could induce expression of transglutaminase 2 (TGase2) at the mesangial surface, which upregulates CD71 and triggers an inflammatory feedback loop through enhanced expression and mesangial cell proliferation as well as inflammatory factors. These data indicate that interactions among IgA1, sCD89, CD71, and TGase2 in mesangial cells are required for IgAN progression.

A previous study demonstrated that the aberrantly glycosylated IgA from IgAN patients contained higher molecular weight pIgA than the normal IgA1 from healthy individuals. And more importantly, the serum IgA immune complexes from patients with IgAN bound more strongly to CD71, but soluble CD71 could significantly block this binding (22). It has been suggested that deposited pIgA1 or IgA1 immune complexes could initiate a process of auto-amplification involving the hyperexpression of CD71, allowing increased IgA1 mesangial deposition of pIgA1 or IgA1 immune complexes may trigger the self-amplification, which correlates to upregulation of CD71, thereby prompting IgA1 deposits in glomerular mesangium (23). Nevertheless, the novel findings in a recent study should be considered seriously (24), as the authors found that human IgA1 can cause glomerular damage in people without significant glycosylation defects, as long as serum levels are high enough or affinity is immature. Similarly, they observed that human IgA1 induced glomerular lesions independent of the IgA receptor CD89 molecule. These findings still need to be verified by more research.

The deposition of O-galactosylated IgA1 immune complexes on the mesangium activates mesangial cells to release inflammatory factors and complements. The activation of the inflammatory cascade responds to podocyte and tubule interstitial damage as well as renal fibrosis through crosstalk among glomerulus-podocyte-tubular epithelial cells (25). Podocytes are considered an important hub between the glomerulus and tubulointerstitial cells (25). Previous studies have confirmed that the mesangial cells incubated with supernatant of co-cultured IgA1 from sera of patients with IgAN can promote the expression of TNF-α and its receptor, and increase the secretion of IL-6 in podocytes (26). PIgA1 triggers mesangial cells to secrete cytokines (such as TNF-α and TGF-β), thereby activating podocytes and causing high permeability of the glomeruli and proteinuria. Simultaneously, the cytokines secreted by mesangial cells also stimulate the tubulointerstitial cells to produce other inflammatory factors or chemokines, leading to renal tubular damage, interstitial inflammatory cell infiltration and fibrosis (25). The TNF-α released by mesangial cells can not only stimulate podocytes to secrete TNF-α in an autocrine manner but also upregulate podocyte secretion of TNF-αR1 and TNF-αR2. Additionally, when TNF-α combines with TNF-αR1, it will increase IL-6 secretion and induce podocyte apoptosis and renal tubular atrophy (26, 27). It has been reported that IgA1 from blood of IgAN patients stimulates mesangial cells to produce some substances, promoting synthesis of TGF-β via activation of the renin-angiotensin system in podocytes, which then causes renal interstitial fibrosis (28). In addition, mesangial cells also release aldosterone, inducing apoptosis of renal tubular epithelial cells, which leads to tubular atrophy (29).

## Different Perspectives on the Involvement of Mucosal Immune Response in the Pathogenesis of IgAN

The mucosal immune response has been shown to be important in the pathogenesis of IgAN (30–33). It has long been found that during mucosal infection, hematuria and proteinuria are increased in patients with IgAN (34–36), and some cases have an episode of upper respiratory infection at onset (37, 38). Thus, this abnormal mucosal immune response is thought to be related to the occurrence and development of IgAN.

Since the concept of mucosal immunity was proposed in the 1960s, the mucosal immune system (MIS), as a relatively independent immune system in the body has been a concern. More than 95% of infections occur in or from the mucosa. Mucosa-associated lymphoid tissue (MALT) is present in the induction area that forms lymphoepithelial tissue. In these areas, mucosal IgA production is induced in a T-cell-independent and T-cell-dependent manner (39, 40). Primed B cells move to mucosal lamina propria, where they synthesize polymeric mucosal sIgA1 with poor O-galactosylation and low affinity; they are secreted to the mucosal surface but rarely enter the circulatory system. In contrast, most circulating IgA1 produced by bone marrow intrinsic plasma cells is monomeric and heavily O-galactosylated (41, 42).

The current hypothesis is that persistent exogenous antigen stimulation and mucosal-bone marrow axis dysfunction cause the production of pathogenic IgA1 and deposition in glomerular mesangial area, leading to the onset of IgAN (43, 44). The increased level of serum IgA1 with poor O-galactosylation in IgAN may be due to “wrong transfer” to the bone marrow due to the misexpression of homing receptors on the surface of B/plasma cells secreting IgAl in the mucosa, rather than homing to the mucosal surface, resulting in the direct release of “mucosal IgA” into the systemic circulation (45–47).

### Role of the Tonsils in IgAN Pathogenesis

The first study on the relationship between tonsils and IgAN was carried out by Tomino in 1983 (48). It has been demonstrated that the antibodies eluted from the renal tissues of patients with IgAN specifically bound to tonsillar cells, and this effect was entirely suppressed by the addition of anti-human IgA antisera. It has also been reported that the serum IgA levels were increased in about half of patients with IgAN, but levels decreased after receiving a tonsillectomy, suggesting a relationship between tonsils and IgAN (49). Increased levels of circulating immune complexes have been observed after tonsil provocation tests in patients with IgAN complicated by chronic tonsillitis (50). However, following a series of studies, the Japan Society of Stomato-Pharyngology (51) officially analyzed the relevant literature on the clinical benefits of tonsillar provocation test to patients with IgAN and suggested that it lacked value in determining indication for tonsillectomy in patients with IgAN. Nevertheless, the worse urinary findings after tonsillar irritation may be distinct, indicating that tonsil and kidney diseases are related (52). *Haemophilus parainfluenzae* is one of the common bacteria in tonsils. It has been reported that the antigen and antibodies of this bacterium are present in the glomerular mesangium and sera of IgAN patients, suggesting that *H. parainfluenzae* infection may contribute to the pathogenesis of IgAN (53). Another study also found that tonsil mononuclear cells in patients with IgAN can release high levels of IgAl after stimulation with lipopolysaccharide (LPS) and *Streptococcus hemolyticus*, and this effect may be due to its activation-induced cytidine deaminase and increased Iα-Cα expression (54). A single-center controlled study confirmed that the amount of interfollicular monocyte-derived dendritic cells (DCs) in the palatine tonsils is associated with the formation of crescents in IgAN, indicating that DCs in the interfollicular region of tonsils are involved in the course of glomerular vasculitis (55). Analysis in a group of patients with IgAN with gross hematuria found that the expression of chemokine receptor CX3CR1 in the circulating leukocytes was up-regulated, which was consistent with the results from whole-genome sequencing of these patients (56). This finding was supported by another study, which presented that CD8+ lymphocytes from patients with IgAN expressed significantly increased levels of CX3CR1, but this expression declined after tonsillectomy, accompanied by the remission of hematuria. Therefore, the excessive immune response to microbial DNA raises the expression of CX3CR1 of CD8+ lymphocytes in the tonsils of patients with IgAN, subsequently these lymphocytes transfer to renal glomeruli, giving rise to renal damage and hematuria (57). All these findings suggest that local mucosal infection of the tonsil is related to the pathogenesis of IgAN. In light of the above findings, in Asia (especially in Japan), tonsillectomy is generally considered to be beneficial for effective control of IgAN (58). Many studies have claimed that tonsillectomy had a favorable effect on long-term kidney function in patients with IgAN (59–63). A Chinese single-center cohort study found increased frequencies of memory B cells in the blood and tonsils in IgAN (64), but following tonsillectomy, these frequencies were significantly decreased. The author speculated that this may be related to the aberrant mucosa-bone marrow axis in IgAN patients, and IgAN has a Th2-related immunopathogenesis because memory B lymphocytes are mainly involved in the humoral immune response. A meta-analysis of non-randomized studies including 858 Asian IgAN patients that underwent tonsillectomy or not, suggested that the overall clinical remission rate was higher in the patients that received a tonsillectomy. Meanwhile, more patients who did not undergo surgery developed ESRD. The best clinical remission rates occurred in patients who received tonsillectomy combined with steroid pulse therapy. However, it is worth noting that the clinical remission rate of tonsillectomy alone is not superior to that of conventional treatment (65). A recent study from a Japanese group suggested that the timing of tonsillectomy and steroid pulse therapy is very important for the effect on IgAN. It is recommended that tonsillectomy be performed in a short window before and after steroid pulse therapy, which may be helpful for improving IgAN. The potential mechanism is that a steroid pulse can temporarily destroy the tonsil germinal center, causing tonsil atrophy. Before reconstructing the germinal center and restoring proliferation of local immune cells, tonsillectomy may be more conducive to enhancing the effect of therapy (66).

Numerous studies have sought to find a pathogenic correlation between tonsils and IgAN, however, the results are often contradictory. The ability of tonsillectomy to alleviate disease has rarely been observed in European cohorts. In a study involving a large European cohort of patients with IgAN, there was no improvement in IgAN progression after tonsillectomy (67). Furthermore, tonsillectomy did not influence the activation of innate immunity, even though the aberrant galactosylation of IgA1 was obvious in patients with IgAN. The research hardly encourages the involvement of tonsillectomy in the modification of mucosal immune response in patients with IgAN, but implies that activation of the gut mucosal immune network may potentially contribute to glomerulonephritis (68). This may indicate that the role of tonsils in IgAN pathogenesis is not significant enough, at least not in the numerous patients outside Asia. The reasons may involve the influence of genetics, environmental factors, attitude and timing of kidney biopsy, and other factors (39, 69–72). In Japan and China, early screening including renal biopsy is being used more aggressively, and even routinely in some centers.

### Role of Intestinal Mucosal Immune in IgAN Pathogenesis

In recent years, the role of commensal microorganisms (microbiota) colonized on the surface of human intestinal mucosa, in both healthy and diseased conditions, has attracted attention. The microbes on the mucosal surface are in close contact with the intestinal epithelium and exert a significant effect on the modulation of intestinal mucosal immune by influencing the intestinal barrier against pathogens and the host immune system (73). Under normal circumstances, when the intestinal mucosa is stimulated, intestinal B cells switch from secreted IgM to secreted IgA1. SIgA1 and other secretory components (such as antimicrobial peptides and mucus secreted by Paneth cells and goblet cells, respectively) synergetically play a host defense role against the invasion of pathogens on the mucosa (74). Altered intestinal barriers might facilitate an abnormal response to microbiota, triggering MALT activation and subclinical intestinal inflammation (75). This can induce an abnormal response to alimentary antigens or commensal microbes, along with the synthesis of poorly glycosylated pIgA1, which eventually deposits in the mesangium through circulation.

In patients with IgAN, several clinical observations have shown activation of the innate immune system on the intestinal mucosa (76–78). A link between IgAN and genes concerned with the immunity of intestinal pathogens has been reported in a genome-wide association study (GWAS). The results of the GWAS support the hypothesis that genetic risk, geospatial risk, and environmental variables may collectively induce alteration of the gut mucosal immunomodulation, which facilitates the course of IgAN (30). A clinical investigation from China (79) found that chronic enteritis was the second most common mucosal symptom of IgAN patients after chronic pharyngitis, with 35.2% of patients with IgAN presenting with chronic enteritis simultaneously. The incidence of chronic enteritis in patients with hematuria was markedly higher than that in patients without hematuria, indicating that gastrointestinal mucosal immunity is also closely related to IgAN.

It has been demonstrated that increased bacterial LPS exposure is related to poorly galactosylated IgA. B cells stimulated by LPS can promote the methylation of the chaperone Cosmc, thereby downregulating the expression of the Cosmc gene, which is the basis of abnormal O-glycosylation in the IgA1 hinge region of patients with IgAN, and thus promotes the formation of poorly galactosylated IgA (80). Other experimental models suggest the involvement of alimentary antigens (e.g., gluten) (81, 82). Increased IgA levels in response to alimentary antigens has been observed in the peripheral blood, which is correlated to increased intestinal permeability (83, 84). Coppo et al. (82) successfully established a murine model of gluten-induced experimental IgA glomerulopathy. Following this report, Papista et al. studied the relationship between gluten and IgA deposition in a spontaneous IgAN murine model expressing human IgA1 and CD89 (85). The mice were fed a gluten-free diet for three generations, and they observed an improvement of mesangial IgA1 deposits, alleviation of glomerular inflammation, and reduction of IgA1-sCD89 immune complex levels in the blood and renal eluates. On the contrary, a high-gluten diet could induce glomerular inflammation and IgA1-sCD89 complex deposition. In combination with the finding that high levels of anti-gliadin IgA are closely associated with the degree of proteinuria in patients with IgAN, a gluten-free diet may be useful in ameliorating the development of IgAN. All the above findings suggest that the gut mucosal immune system of patients with IgAN is hyper-reactive to mucosal antigens, such as alimentary antigens or microbial components.

## Innate Immunity and IgAN

### Role of Toll-Like Receptors in IgAN Pathogenesis

It is well-known that the mucosal surface is the main site of innate immunity. As mentioned above, the innate immune system works through the recognition of pathogen- and damage-associated molecular patterns (PAMPs and DAMPs, respectively) induced by macrophages, DCs, leukocytes, and other cells. The system increases opsonification and phagocytosis, which facilitate the swift elimination of pathogens. Toll-like receptors (TLRs) are the key components of the mammalian innate immune system and mediate immune and inflammatory responses through binding PAMPs and/or DAMPs (86). Myeloid differentiation factor 88 (MyD88) is the adaptor for TLR-mediated signal transduction (87).

Antigen components from air, food, microorganisms, or necrotic cells are recognized as a DAMP or PAMP by TLRs on the surface of the mucosa, thereby initiating the innate immune response (88). Upon activation, TLRs initiate an intracellular signaling cascade, the release of cytokines, and the enhanced expression of cell surface costimulatory molecules (89). TLR activation induces DC maturation, chemokine release, and recruitment of inflammatory cells in the infected site. Subsequently, the mature DCs move to the lymph nodes, where they interact with T cell receptors (TCRs) of T cells and activate specific T cells and antibody synthesis (90). Consequently, infection can exacerbate kidney inflammation in diverse pathways by activating TLRs. Additionally, inflammatory products can also activate TLRs expressed on intrinsic renal cells, which can further trigger inflammation and tissue impairment. TLRs are expressed not only on circulating immune cells and in infiltrating macrophages/DCs, but also on resident renal cells (91), and they have been considered as mediators of renal diseases, playing a role in DC maturation and in autoimmunity (92). There has been a series of evidence supporting the involvement of TLRs in the pathogenesis of IgAN.

TLR4 recognizes LPS in gram-negative bacteria. It has been observed that the expression of TLR4 mRNA and protein in renal tissue is significantly increased in IgAN rats treated with oral and intravenous immunization of bovine serum albumin (BSA) for 12 weeks (93). The elevated TLR4 expression on monocytes of peripheral blood in patients with IgAN was significantly associated with proteinuria or clinical activity (94). Previous studies have mentioned that activation of TLR4 triggered by bacterial LPS promotes Cosmc methylation, thereby reducing the degree of glycosylation of the IgA1 molecule, which is also the basis of the pathogenesis of IgA nephropathy (80). In an *in vitro* co-culture system of IgA and mesangial cells, TLR4 mediates MAPK activation and MCP-1 secretion, indicating that TLR4 is engaged in glomerular mesangium damage by inducing inflammatory cytokines in IgAN (95). It has been confirmed that TLR4 is involved in the activation of NF-κB. The nuclear translocation of NF-κB triggers the transcription of mRNA encoding many inflammatory mediators, such as cytokines, chemokines, fibrinogen, etc., which contribute significantly to the effects of the innate and adaptive immune responses (96). For example, the nuclear translocation of NF-κB is conducive to B cell proliferation, thus increasing the synthesis of IgA (97). Animal experiments showed that TLRs are involved in the conversion of B cells from IgM to IgA (97). In addition, TLR4 is constitutively expressed in podocytes. Podocytes responding to immune complex-mediated glomerular filtration barrier damage will upregulate the expression of TLR4, thereby resulting in the local release of chemokines, which may allow the recruitment of inflammatory leukocytes and exacerbate glomerular damage (98). Recently, renal TLR4-mediated and profibrotic signaling have been demonstrated in chronic kidney disease. TLR4 expression was significantly associated with the expression of TGF-β1 and altered susceptibility of renal cells to TGF signaling (99, 100).

TLR9 has been characterized as the receptor of viral and bacterial cytosine-phosphate-guanosine (CpG)-DNA. A previous study reported that the IgAN-susceptible ddY mice fed normally showed enhanced transcriptional levels of TLR9 and MyD88 in the splenocytes. These mice treated with nasal CpG-oligodeoxynucleotides can induce Th1 polarization, IgA expansion in circulation and mesangium, and exhibit exacerbated renal lesions. In the same study, TLR9 polymorphism was found to be involved in the development, but not occurrence, of human IgAN, revealing that an analogous mechanism relevant to TLR9/MyD88 may play a role in murine and human IgAN (101). The latest study found that CpG-ODN activates TLR9/MyD88 signaling and induces the production of glycosylated IgA1 and IgG-IgA ICs through the IL-6 and/or a proliferation-inducing ligand (APRIL) pathway, and further aggravates kidney damage. B cells and DCs are directly or indirectly involved in the process of inducing the excessive production of abnormally glycosylated IgA1, respectively (102). A non-randomized controlled study in Japan investigated expression of tonsillar TLR9 in a group of patients with IgAN patients who received tonsillectomy in combination with steroid pulse therapy (103). The results indicated that elevated tonsillar TLR9 levels existed in 22.4% of patients, and TLR9-positive patients showed better response to treatment, reflected in more effective alleviation in urine findings. This indirectly reflects the involvement of TLR9 in IgAN development. Additionally, up-regulation of TLR 4, TLR 7, TLR 8, and TLR 9 has also been observed in the renal tissues of patients with IgAN (104). A real-time RT-PCR analysis suggested the mRNA expression of TLR2, TLR3, TLR4, TLR5, TLR7, and TLR9 were markedly increased in the peripheral blood mononuclear cells of patients with IgAN. Among them, more obvious proteinuria was present in the patients with high mRNA levels of TLR2, TLR3, TLR5, or TLR9, while increased serum IgA was observed in patients with high mRNA levels of TLR4 (105). The above data suggest that PAMPs and/or DAMPs can aggravate glomerular inflammation in various ways by activating TLRs. In addition, inflammatory mediators can also activate TLRs present on the renal intrinsic cells, which can further trigger inflammation and tissue damage (90).

### Role of Complements in IgAN Pathogenesis

Based on laboratory evidence and results of renal biopsy in patients with IgAN, it is known that complements play important roles in IgAN; however, the specific role of complement activation in the mechanisms of disease is still not fully clear. The complement system consists of the activation cascade of ~50 proteins located in the plasma, tissues, and cells, including complement component proteins (C3, C5, etc.), complement regulatory proteins (factors H, I, etc.) and complement receptor proteins (CRl, CR2, etc.). There are three commonly accepted complement activation pathways, including the classical pathway (CP), the alternative pathway (AP), and the lectin pathway (LP). Each pathway has a respective triggering mechanism initiated by the interaction of complement proteins with distinct structures.

In IgAN, one of the characteristic manifestations is that IgA is generally co-deposited in mesangium with complement proteins. Immunohistochemical evidence shows deposits of C3, complement factor P (CFP), C4d, mannose-binding lectin (MBL), and membrane attack complex (MAC) in the mesangium of IgAN, along with the lack of C1q usually. Although some studies showed that glomerular mesangial C1q deposition in patients with IgAN was associated with poor renal prognosis and serious pathological features, this finding has not been generally accepted (106, 107). C1q is rare in the renal tissue of patients with IgAN (108), and its presence and clinical significance in IgAN are still inconclusive. AP is considered the most important effect pathway in the pathogenesis of IgAN. It has been proposed that pIgA, aggregated IgA, and abnormally glycosylated IgA have a powerful ability to activate AP (109, 110). *In vitro* assays using IgA coated directly on plastic surfaces, or serum pIgA showed that IgA1 is capable of triggering the cascade and cleaving C3 through AP (111, 112). Evidence for AP-dependent complement activation and essential effector molecular regulation of IgAN suggests that a gd-IgA1-immunocomplex can be used as a complement activation surface.

The co-deposition of C3 with IgA1in the mesangium is correlated with the severity and progression of IgAN. Previous studies have shown that poorly glycosylated pIgA1 in the peripheral blood can bind with IgA-binding M protein from *Streptococcus pyogenes* serotype M4 and co-deposit in the mesangium, which enhances the synthesis and secretion of IL-6 and C3 as well as the proliferation of mesangial cells, thereby promoting the inflammatory progression of IgAN (113). Besides C3 deposition in the kidney, lower C3 with higher C3 cleavage (iC3b and C3d) is detected in the peripheral blood of some patients with IgAN, suggesting systemic complement activation (114). A Korean observational cohort study involving a total of 343 patients with biopsy-proven IgAN showed that serum C3 and glomerular mesangial C3 deposits are independently associated with poor prognosis of IgAN (115). Similarly, a Chinese single-center cohort study analyzed the correlations of serum/urinary C3a and C5a with clinical manifestation and histopathology in patients with IgAN. They found that C3a and C5a depositions in IgAN were obviously enhanced, and they increased with the exacerbation of pathological impairment (116). In addition, another study found that the IgA/C3 ratio in the peripheral blood shows a significant positive correlation with quantities of protein in the urine of patients with IgAN (117).

Several previous reports indicated that AP complement components (factors B and P, CFB, and CFP) and complement regulatory protein (complement factor H, CFH) are widely present in the kidney tissues of patients with IgAN, and that there are also significantly increased CFB and CFP levels in the circulation of patients with IgAN (118, 119). CFH is the most important regulatory protein in AP, whose main function is to make the complement system clear pathogens or other dangerous substances, but not impair their own tissues. In addition to CFH, there is also a group of proteins that shows high sequence homology with CFH, namely complement FH-related proteins (CFHRs, including CFHR-1, CFHR-2, CFHR-3, CFHR-4, and CFHR-5). Functionally, CFHRs deregulate CFH by competing with CFH to bind C3b and promote activation of AP (120).

A GWAS of IgAN in Chinese and European cohorts found that a CFH gene variant on chromosome 1q32 (*rs6677604*) is associated with IgAN (121). The same team also found that the *rs6677604-A* allele is involved in increased CFH and decreased C3a levels in serum (122). Moreover, serum C3 levels negatively correlated with mesangial C3 levels, whereas CFH levels were positively correlated with serum C3 levels. Decreased or absent CFHR1/CFHR3 levels further weakened the deregulation effect of CFH. This synergistic effect increased the negative regulation of complement activation caused by aberrant glycosylation of IgAl in IgAN, which may influence the formation of circulating immune complexes, thereby reducing risk of IgAN occurrence. It has been reported that in patients with IgAN, CFH levels were normal, but CFHR-1 levels were enhanced (123), and increased CFHR-1/CFH ratios were consistent with impaired renal function. In IgAN pathogenesis, CFHR-1 antagonizes CFH, thereby inhibiting the activation of C3. A study from another group also came to a similar conclusion (124).

In addition to CFH, CFHRl, and CFHR3, the role of CFHR5, another complement FH-related protein, has attracted extensive attention regarding its role in the pathogenesis of IgAN. A prospective clinical study showed that glomerular FHR5 deposition existed in all patients with IgAN, just in the same manner that C3 and sc5b-9 did (125). Recently, a study of a large Chinese cohort suggested that *CFHR5* is an IgAN predisposition gene (126), and another study also indicated that serum CFHR-5 level was independently correlated with IgAN progression (127). It has been proposed that FHR1 and FHR5 homodimers can disturb the physiological effects of FH. In addition, aberrant FHR levels may trigger and promote AP activation, resulting in complement cascade-associated inflammation and renal lesions (128).

In IgAN, different research teams found that about 25% of patients with IgAN exhibited positive renal local MBL staining, suggesting the presence of complement activation of LP (129). MBL, L-ficolin, M-ficolin, and H-ficolin are all complement-activating soluble pattern recognition molecules, which interact with PAMPs and/or DAMPs and initiate complement activation through MBL-associated serine protease (MASP)-1, MASP-2, and MASP-3, activating C4 and C2 and leading to C4b2a formation and C3 cleavage. LP activation on the surface of pathogens plays a first-line role in host defense (130). Clinical analysis showed that M-ficolin, L-ficolin, and MASP-1 were elevated, whereas MASP-3 levels were reduced in the blood of patients with IgAN, indicating that MASP-3 levels may be related to IgAN severity (131). The abnormal glycosylation of IgA1 is characterized by high levels of GalNac exposure, which may interact with ficolins to activate LP (132). In the absence of CP activity, MBL-MASP causes C3 and C4 to deposit on immobilized IgA, suggesting that a dose-dependent binding of MBL can bind pIgA, but not monomeric IgA, and activate complement LP (133). It has been reported in IgAN that positive renal local MBL staining and urinary MBL levels are associated with poor prognosis, suggesting that complement activation of LP is involved in IgAN pathogenesis (134, 135). In addition, the MBL level of patients with IgAN was closely related to the MBL2 genotype. In patients with IgAN, the incidence of prodromic infection and gross hematuria is significantly higher in individuals with MBL deficiency, and the long-term prognosis is poor. The possible underlying mechanism is that the risk of infection is increased in patients with MBL deficiency, and infection may induce impaired mucosal immune regulation, which promotes the progression of IgAN (136). The findings of many studies have suggested that the activated fragments of C4 deposited in the glomerular mesangium of patients with IgAN are more likely to be produced by the MBL pathway (137, 138). Mesangial deposition of C4d has been identified as a significant risk factor for worsening renal function (139–141). A retrospective study on renal biopsies of 15 cases of IgAN showed that segmental and global deposition of C4d was particularly associated with endocapillary proliferation; moreover, interstitial fibrosis and tubular atrophy were more severe in C4d-positive patients with IgAN (142).

In addition to AP and LP, it is worth noting that the complement terminal pathway also is involved in IgAN pathogenesis. Treatments targeting this pathway are starting to be used clinically. As early as the 1980s, studies on histopathological analysis of IgAN biopsies began to demonstrate glomerular C5b9 deposition (143). Using immunofluorescence, researchers quantified MAC, properdin, CFH, and complement receptor type 1 (CR1, an inhibitor of the complement system) in urine samples of patients with IgAN (144). There was glomerular deposition of C5, CFH, and properdin; urinary MAC, CFH, and properdin levels were obviously elevated, but CR1 levels were apparently reduced. A proteomics study of IgAN kidney biopsy specimens from Norway showed an increased abundance of proteins of the terminal complement pathway, suggesting complement-mediated impairment in progressive IgAN. Meanwhile, one study reported a lower abundance of CR1 in progressive IgAN, which may reflect an underlying mechanism involved in reducing complement inhibitory control in IgAN (145).

## Adaptive Immunity and IgAN

Adaptive immunity is certainly correlated with IgAN pathogenesis. Under physiological circumstances, as previously mentioned, T cells activated by DCs presenting mucosal antigens (bacterial or viral products, alimentary antigens, etc.) trigger naive B cells to undergo IgM-to-IgA class switch in a T-cell-dependent manner in Peyer's patches and tonsils. Activated B cells migrate to regional lymph nodes and home to MALT, where they eventually differentiate into IgA-secreting plasma cells. In addition, IgA can be produced in a T-cell-independent manner in the MALT. The IgA dimers move through the mucosal epithelium to become SIgAs, which act in defense against pathogens. In the pathogenesis of IgAN, the continued exogenous antigen stimulation, abnormal mucosal immune response, and the incorrect expression of homing receptors on the surface of IgAl-secreting B/plasma cells can result in elevated aberrantly glycosylated IgA1, which tends to self-aggregate into pIgA1. Meanwhile, glycosylation deficiency brings about GalNAc exposure and is recognized by natural IgG and IgA1 antibodies as an antigen, thereby resulting in the formation of immune complexes. Furthermore, soluble CD89/Gd-IgA1-IgG complexes are deposited in the mesangium by binding to their receptors and continuous release of cytokines and growth factors in the local mesangium, leading to inflammatory damage, matrix accumulation, and glomerulosclerosis. Finally, further recruitment of T lymphocytes leads to renal tubular interstitial damage and fibrosis (146).

### Role of T Cells in IgAN Pathogenesis

Multiple T-cell subsets, especially Tc, Th2, Th17, Th22, T follicular helper (Tfh), and regulatory T-cells (Tregs), are major contributors to the pathogenesis and pathophysiology of IgAN. Th1 lymphocytes are primarily involved in cell-mediated immunity, secreting IFN-γ, IL-2, TNF-β, and facilitating the elimination of intracellular pathogens, whereas Th2 lymphocytes regulate humoral immunity and mediate the clearance of parasites by producing IL-4, IL-5, IL-9, and IL-13. It is generally believed that patients with IgAN have Th1/Th2 imbalances that tend to favor Th2 shift (147–149). Previous study had shown that the ratio of Th1/Th2 in tonsil lymphocytes in IgAN patients with tonsillitis was lower than that in patients with common chronic tonsillitis, and Th1/Th2 ratio was consistent with proteinuria and histopathology features in IgAN group (147). It has been observed that Th2 differentiation promoted poor galactosylation of IgA in a murine model of IgAN (148). Subsequent studies demonstrated that IL-4 treatment resulted in decreased expression of C1GALT1C1 mRNA of B cells from patients with IgAN, by methylation modification, which is beneficial to the secretion of aberrantly glycosylated IgA1 (149). Moreover, the response of glomerular cells to Gd-IgA immunocomplexes could be enhanced by Th2 lymphocytes, thereby reducing the glomerular filtration rate (GFR) (148). In addition, as a Th1-type cytokine, IFN-γ may be beneficial for preventing the progression of IgAN (150). Some studies also reported that IFN-γ serum levels were rarely significantly increased in patients with IgAN compared with the obvious elevation of Th2-type cytokines (151, 152). Moreover, as another major cytokine secreted by Th1 cells, the level of IL-2 has no correlation with serum IgA levels, the severity of renal histological changes, or other clinical parameters in patients with IgAN (153). Additionally, it should be emphasized that IL-2 is not unique to the Th1 subgroup. Other Th subgroups, activated Tc cells, NK T cells, and dendritic cells also can secrete a large amount of IL-2 (154). It has been shown that Th1 cells may be predominant during the late stage of IgAN with crescent formation and tissue damage (155). Another Italian study of whole-genome DNA methylation screening in CD4+ T cells in patient with IgAN proposed that there are specific methylation abnormalities in CD4+ T cells in patients with IgAN, which may cause reduced TCR function and decreased T cell activation, thereby leading to a Th1/Th2 imbalance with an enhanced IL2/IL5 ratio. These abnormalities may be related to the subsequent immunoglobulin class-switch to IgG production (156).

Tfh cells were first discovered in human tonsils and described as a subset of CD4+ T cells expressing the chemokine receptor CXCR5. They are localized in the B-cell region of lymphoid tissues and play a pivotal function in the formation of memory B cells and long-lived plasma cells (157). It was reported that there is a higher frequency of CD4+CXCR5+ Tfh cells in patients with IgAN, and there is a negative correlation between this subpopulation and estimated GFR. In addition, there are positive correlations among CD4+CXCR5+PD-1+ Tfh cells and serum IL-21, Gd-IgA1, and proteinuria. This indicates that high Tfh frequency may be involved in the development of IgAN (158). Another study indicated that Tfh might be involved in IL-21-mediated production of IgA and Gd-IgA1 (159). The study also found that IL-21 promoted the level of activation-induced cytidine deaminase in B cells, thereby promoting somatic hypermutation. According to a previous study, anti-Gd-IgA1 autoantibodies originate from somatic hypermutation of heavy-chain gene segments in anti-Gd-IgA1-producing cells (160).

Th17 and Treg cells are frequently found on the mucosal surface, where they implement their defenses against microbial invasion and prevent excessive immune responses, respectively. It has been identified that Th17 cells, driven by IL-23, are involved in autoimmune inflammation (161), whereas Tregs suppress the immune responses and inflammation, and maintain peripheral tolerance. Studies have shown that these two T-cell subsets can be transformed into each other under certain conditions. RORγt and Foxp3 are the main modulators of Th17 cells and Tregs, respectively, and the balance of their expression levels determines the direction in which T-cell subsets differentiate (162). The balance of Th17 and Treg cells *in vivo* maintains immune homeostasis (163).

It has already been demonstrated that patients with IgAN present a reduced frequency of CD45RA(-) FoxP3 (high) activated Treg subset, elevated frequency of Th17, as well as correspondingly elevated serum levels of Th17-associated cytokines, such as IL-17A, IL-21, IL-23, IL-1β, and IL-6, and decreased serum levels of Treg-associated cytokine, IL-10. The patients with high IL-17A expression had lower renal function, greater proteinuria, and more severe tubulointerstitial damage (164). Upon IL-17 stimulation, B lymphocytes proliferate and lead to increased production of underglycosylated IgA1 *in vitro* (165). Moreover, mesangial cells stimulated by Gd-IgA1 are capable of producing CCL20, and therefore, inflammatory Th17 cells recruited to the kidney induce further glomerular lesions in IgAN (166). Previous studies suggested that the frequencies of Tregs in the peripheral blood and tonsils of patients with IgAN were significantly lower than those in the healthy controls. The reduction is primarily ascribed to the decreased levels of induced Tregs, but no significant alteration in levels of natural Tregs is observed (167, 168). In addition, another study revealed not only a deficient quantity but also poor immunosuppressive function of Tregs in IgAN. Perhaps because of the dual defect in both quantity and function, Tregs of patients with IgAN hardly prevent formation of Gd-IgA1 mesangial deposits, and subsequent infiltration of inflammatory cells as well as proliferation of the mesangial matrix (169). T cell development is regulated by microRNAs (miRNAs) post-transcription. As one of the post-transcriptional regulator of miRNAs, miR-155 can regulate mammalian immunity in various ways. It has been demonstrated that peripheral blood lymphocytes of patients with IgAN express extremely low levels of mir-155. This causes a T-cell subset shift that makes individuals prone to the development of autoimmune diseases (i.e., elevated frequencies of Th2 and Th17 along with reduced frequencies of Th1 and Treg), which is beneficial for inhibiting the expression of Cosmc gene and aggravating poor IgA1 glycosylation (151).

Concerning the role of Tc cells in the pathogenesis of IgAN, it has been previously mentioned that the high expression of CX3CR1 is presented in the peripheral blood CD8+ Tc of patients with IgAN, which boosts lymphocytes moving across the glomerular endothelium and causes glomerular capillary wall destruction and hematuria (56, 57).

### Role of B Cells in IgAN Pathogenesis

B cells are essential in the early stage of IgAN pathogenesis. The existing data indicate that besides TLRs, the mucosal response of B cells also involves APRIL and B-cell activation factor (BAFF), which makes an important contribution in IgAN progression (170, 171). APRIL and BAFF are two important factors for B cell homeostasis; they share two receptors–transmembrane activator and calcium modulator cyclophilin ligand interactor (TACI) and B cell maturation antigen (BCMA), and BAFF also has a unique receptor: BAFF receptor (BAFF-R) (172). APRIL synthesis is enhanced when pathogens on the mucosal surface induce TLR9 expression, which further contributes to T-cell-independent IgA class switching on B cells. One study showed that TLR9 stimulation can induce abnormal expression of APRIL in B cells of the tonsil germinal center of patients with IgAN (173). APRIL promotes increased production of IgA, mainly Gd-IgA1. The findings from a controlled study showed that the expression of APRIL and its receptors is elevated in B cells from patients with IgAN, which promotes the hypersecretion of Gd-IgA1 (174). A recent study identified that over-production of IgA from tonsillar mononuclear cells of patients with IgAN is induced by the APRIL pathway and that high expression of TACI on patients' B-cells induced by oligodeoxynucleotides with CpG will promote APRIL-associated IgA production (175). In a group of patients with IgAN recurrence after renal transplantation, the serum levels of APRIL remained relatively high for nearly 3 years after surgery (176). Moreover, it has been demonstrated that IL-6-mediated overproduction of Gd-IgA1 can be entirely inhibited by treatment with APRIL-specific siRNA in ddY mice (102).

BAFF is involved in B cell survival, maturation, proliferation, and differentiation. During mucosal infection, myeloid cells secrete IL-12, IL-8, and IFN-α, and these cytokines boost the secretion of IFN-γ by NK cells, which further up-regulates the expression of BAFF. Excessive BAFF levels hinder B cell proliferation and differentiation into antibody-secreting cells, promoting the production of aberrantly glycosylated IgA1. Several groups have previously measured BAFF expression in IgAN. Treating tonsillar mononuclear cells from IgAN patients *in vitro* by physical or chemical stimulation may activate BAFF, and induce secretion of aberrant O-glycosylated IgA1 by suppressing the expression of C1GALT1 and Cosmc (177, 178). It has been identified that elevated levels of TLR9 and BAFF are conducive to the overexpression of serum IgA1 and associated with renal function and disease activity of IgAN (179). Interestingly, the findings from a clinical cohort study proposed that *Streptococcus pyogenes* infection was closely associated with BAFF production in patients with IgAN, but the levels of BAFF were decreased (172). In addition, a study of comprehensive transgenic animal models and clinical data indicated that although BAFF-overexpressing Tg mice can induce IgAN, peripheral blood in patients with IgAN revealed a unique elevation of APRIL levels and no significant change in BAFF levels (180). Martín-Penagos et al. hypothesized that this phenomenon may have occurred because TACI expressed in B cells in the human small intestine upregulates APRIL-induced IgA synthesis and downregulates BAFF-mediated responses *in vitro*. Upon binding to TACI, APRIL is more strongly correlated to IgA class switching than BAFF (176).

Evidence from animal models suggests that CD19+ B cells are essential for the pathogenesis of IgAN. Furthermore, the reconstitution of IgAN by transfusion of murine spleen cells without CD90+ pan T cells and transfusion of CD19+ cells in SCID mice indicates that the responsible B cells are involved in nephritogenic IgA synthesis in a T-cell-independent manner (181). A Chinese clinical cohort study demonstrated that the levels of tonsillar CD19+CD5+B cells of patients with IgAN were positively associated with the severity of renal histopathology (182). Another study investigated the features of CD19+CD5+ B cells in the circulation, ascites, and biopsy tissues of patients with IgAN. The results revealed that the frequency of CD19+CD5+ B cells can be observed in above samples of all patients with IgAN. Furthermore, higher levels of IgA and IFN-γ are secreted by CD19+CD5+ B cells in patients with untreated IgAN (183).

## Summary

The most widely accepted “four-hit” hypothesis about the pathogenesis of IgAN implies that immunological factors are engaged in all aspects of IgAN development and play a critical role. Chronic stimulation of harmless antigens, such as bacteria or virus products, alimentary antigens or airborne antigens contributes to abnormal mucosal immunoregulation. In pathological conditions, abnormal expression of effector molecules and disordered activation and differentiation of immune cells in the innate and adaptive immune system synergistically cause poorly glycosylated IgA1 self-aggregation, immune complex formation, and deposition on the mesangium, which further stimulates local inflammation and immune response, causing tissue damage and pathological repair.

Aiming at all aspects of immune mechanism involvement, it is helpful to develop novel and promising clinical early-diagnosis and prognostic indicators, as well as ideal specific targeted therapeutic drugs in translational medicine. In fact, there are already some promising detection indicators and therapeutic approaches in preclinical stages of development. Certainly, we must also be aware that the current research progress on the immunological mechanism of IgAN is incomplete, and sometimes even contradictory. It is still very difficult for us to systematically link all evidence into fully clear pathogenesis. The following work will require continued efforts to systematically understand IgAN.

## Author Contributions

SC is responsible for writing the review. X-KL is responsible for determining the topic and supervising the content of the article.

### Conflict of Interest

The authors declare that the research was conducted in the absence of any commercial or financial relationships that could be construed as a potential conflict of interest.
